# Occurrence, genetic diversity, and antimicrobial resistance of methicillin-resistant *Staphylococcus* spp. in hospitalized and non-hospitalized cats in Brazil

**DOI:** 10.1371/journal.pone.0309711

**Published:** 2024-10-03

**Authors:** Thayanne Gabryelle Viana de Souza, Jordana Almeida Santana, Marina Mourão Sena Claudino, Silvia Trindade Pereira, Rafael Gariglio Clark Xavier, Victor Santos do Amarante, Yasmin Gonçalves de Castro, Elaine Maria Seles Dorneles, Flavia Figueira Aburjaile, Vasco Ariston de Carvalho, Bertram Brenig, Rodrigo Otávio Silveira Silva

**Affiliations:** 1 Veterinary School, Federal University of Minas Gerais, Belo Horizonte, Minas Gerais, Brazil; 2 Clínica Veterinária MedVet, Belo Horizonte, Brazil; 3 Departamento de Medicina Veterinária, Universidade Federal de Lavras, Lavras, Minas Gerais, Brazil; 4 Instituto de Ciências Biológicas, Federal University of Minas Gerais, Belo Horizonte, Brazil; 5 Institute of Veterinary Medicine, University of Göttingen, Göttingen, Germany; Defense Threat Reduction Agency, UNITED STATES OF AMERICA

## Abstract

Methicillin-resistant *Staphylococci* (MRS) cause infections at various sites and exhibit multidrug resistance. Despite their importance in veterinary medicine, only little is known about *Staphylococcus* spp. colonizing and infecting cats. Therefore, in this study, we aimed to isolate and identify *Staphylococcus* spp. colonizing hospitalized and non-hospitalized domestic cats and analyze their antimicrobial resistance profiles, genetic diversity, and risk factors associated with MRS colonization. A total of 218 oral and axillary swabs were obtained from 109 cats, including 77 non-hospitalized and 32 hospitalized cats. After plating on selective media, the isolates were identified via matrix-assisted laser desorption/ionization time-of-flight mass spectrometry and *rpo*B and *16S rRNA* gene sequencing. Subsequently, antimicrobial sensitivity of the strains was assessed, and they were screened for *mec*A gene. Methicillin-resistant *S*. *haemolyticus* (MRSH) isolates were subjected to multilocus sequence typing, whereas methicillin-resistant *S*. *pseudintermedius* (MRSP) and *S*. *felis* isolates were subjected to whole genome sequencing. *S*. *felis* was most commonly isolated from non-hospitalized cats (28.1%), whereas *S*. *pseudintermedius* and MRS were commonly isolated from hospitalized cats (25%). MRSH isolates from hospitalized animals were classified as ST3. The identified MRSP strains belonged to two well-known sequence types, ST551 and ST71. Moreover, antimicrobial use (*p* = 0.0001), hospitalization (*p* = 0.0141), and comorbidities (*p* = 0.002) were associated with increased MRS prevalence in cats.

## Introduction

*Staphylococcus* spp. colonize various hosts and play crucial roles in the oral and skin microbiota of humans and animals [1, 2]. Among more than 75 species identified to date, *S*. *aureus* and *S*. *pseudintermedius* cause infections at different sites, with many methicillin-resistant *Staphylococci* (MRS) detected worldwide, especially in animals and hospitalized individuals [3–6]. MRS are characterized by beta-lactam resistance with low-affinity penicillin-binding proteins (PBP2a or PBP2). One such protein is encoded by the *mec*A gene located on a mobile genetic element, known as staphylococcal cassette chromosome *mec* (SCC*mec*) [7]. MRS are also resistant to other class antibiotics, such as macrolides, aminoglycosides, tetracyclines, and fluoroquinolones [8, 9].

Among the various MRS observed in veterinary medicine, methicillin-resistant *S*. *pseudintermedius* (MRSP) is the most important bacterial agent detected in small animals. MRSP causes hospital outbreaks and severe infections, such as pyoderma, surgical site infections, external otitis, and urinary tract infections [10–13]. Furthermore, these strains act as reservoirs for resistance determinants in other staphylococci, facilitating the spread of multidrug resistance and impacting the efficacy of antimicrobials [10–13]. Many studies have indicated the importance of methicillin-resistant coagulase-negative *Staphylococci*. Methicillin-resistant *S*. *haemolyticus* (MRSH) and *S*. *epidermidis* widely infect humans and animals [14–20].

Despite the importance of MRS in veterinary medicine, their roles in the infection and colonization of cats remain unknown. Cats act as carriers of MRSP and are susceptible to MRSP infections [17, 21–23]. However, risk factors associated with MRS colonization of cats remain unknown. Only a few studies have explored the genetic diversity and resistance profiles of these isolates in cats [17, 22–24], with none focused in Brazil.

In this study, we aimed to isolate and identify *Staphylococcus* spp. colonizing hospitalized and non-hospitalized domestic cats. Additionally, we examined their antimicrobial resistance profiles, genetic relationships, and risk factors associated with MRS colonization.

## Material and methods

### Sample collection

A cross-sectional study using convenience sampling was conducted among non-hospitalized domestic (n = 77, 70.7%) and hospitalized (n = 32, 29.3%) cats at a veterinary clinic in Belo Horizonte, Minas Gerais (Brazil), totaling 218 oral and axillary swabs from 109 cats. Samples from household cats were collected during visits by the research team, whereas those from hospitalized cats were collected from the inpatients at the MedVet Veterinary Clinic in Belo Horizonte, Brazil.

Swab samples were stored in the Stuart Transport Medium (HiMedia, India) under refrigeration (4 °C) for up to 36 h until processing. For both groups, information on sex, age, outdoor access, interaction with other cats or animals, presence of comorbidities, and use of antimicrobials in the last six months was collected (S1 File). This study was approved by the Ethics Committee on Animal Use of the Federal University of Minas Gerais (protocol 287/2019). Informed consent was obtained from all owners through an online consent form prior to sample collection.

### Isolation and identification of *Staphylococcus* spp.

Swab samples were plated on selective media (Mannitol Salt agar, MS–KASVI, Italy) and incubated at 37 °C for 24 h. Colonies formed were subsequently plated on the brain heart infusion agar (Oxoid, UK) and stored in the brain heart infusion broth (Oxoid) supplemented with 20% glycerol at –20 °C. For matrix-assisted laser desorption/ionization time-of-flight mass spectrometry (MALDI-TOF MS), each sample was plated on the brain heart infusion agar (Oxoid), as previously described [12, 25]. Then, 1 μL of formic acid (70%) and 1 μL of MALDI-TOF MS matrix, consisting of a saturated solution of α-cyano-4-hydroxycinnamic acid (Bruker Daltonics, Bremen, Germany), were applied to the spot and allowed to air dry. The spectra were acquired using the FlexControl MicroFlex LT mass spectrometer (Bruker Daltonics). Prior to measurement, calibration was performed using a bacterial test standard (*Escherichia coli* DH5 alpha; Bruker Daltonics). Isolates with MALDI-TOF score < 2.0 were sujected to DNA extraction using the guanidine method to sequence *rpo*B and *16S r*RNA to confirm their identity [26, 27]. Furthermore, isolates belonging to the *S*. *intermedius* group (SIG) were subjected to monoplex polymerase chain reaction (PCR) to detect *nuc* (*pse*), as previously described [12, 28].

### Antimicrobial susceptibility testing

All isolates were subjected to a disk diffusion test as recommended by the M100-Ed31 Clinical and Laboratory Standards Institute guidelines [29]. The following antimicrobials were selected based on previous reports [12, 13]: oxacillin (1 μg), cefoxitin (30 μg), penicillin (10 IU), ciprofloxacin (5 μg), chloramphenicol (30 μg), clindamycin (2 μg), erythromycin (15 μg), gentamicin (10 μg), nitrofurantoin (300 μg), rifampicin (5 μg), sulfamethoxazole-trimethoprim (1.25/23.75 μg), and tetracycline (30 μg) (DME, Brazil). *S*. *aureus* ATCC^®^ 25923 was used as a control. Isolates exhibiting resistance to three or more antimicrobial classes were classified as multidrug-resistant (MDR) strains [30].

### Detection of *mec*A

Isolates resistant to oxacillin or cefoxitin in the disk diffusion method were subjected to DNA extraction using guanidium thiocyanate, as previously described [31]. After quantification using the NanoDrop spectrophotometer (Thermo Fisher Scientific, Wilmington, DE, USA), all isolates were subjected to PCR [32] to detect *mec*A using the following primers: forward 5´-AAAATCGATGGTAAAGGTTGGC-3´ and reverse 5´AGTTCTGCAGTACCGGATTTGC-3´.

### Multilocus sequence typing (MLST) of MRSH isolates

MRSH strains were subjected to MLST, as previously described [14, 33]. Briefly, genomic DNA was extracted using the Wizard Genomic DNA Purification Kit (Promega, EUA), and PCRs was performed as previously described [12, 13]. Sequencing reactions were performed using a BigDye Terminator Cycle Sequencing Kit (Life Technologies) on an ABI 3730XL Genetic Analyzer (Life Technologies). Alleles and sequence types (STs) were analyzed using the PubMLST database for *S*. *haemolyticus* (http://pubmlst.org/shaemolyticus/). Phyloviz v 2.0, using the goeBURST algorithm [34, 35], was used to infer the population structure, with clonal complexes (CCs) composed of all strains sharing at least six identical alleles (single-locus variants). A Neighbor-joining tree was generated from concatenated sequences of the seven housekeeping genes using MEGA version X (https://www.megasoftware.net/) with bootstrap analysis with 1000 replicates, and visualized and annotated using iTOL v.4 [14, 33, 36].

### Genome sequencing of MRSP and *S*. *felis* isolates

Next, three MRSP and six *S*. *felis* isolates were subjected to genome sequencing [12, 13, 37]. The strains were incubated on Mueller–Hinton agar at 37 °C for 24 h. Genomic DNA was extracted using Wizard Genomic DNA Purification Kit (Promega, EUA). Genome sequencing was performed using the Illumina HiSeq platform (mid-out 2 × 150 bp cycles), and the raw data were analyzed using FastQC (Babraham Bioinformatics, Cambridge, England), retaining only paired reads with Phred quality of 30 or higher and a minimal size of 50 nucleotides.

The assembly was performed using SPAdes 3.5.0 in the careful mode [38]. GAP filling and polishing were performed using a Pilon [39]. ResFinder 4.1 [40–42] and PlasmidFinder 2.1 [43, 44] were used to identify the determinants of acquired antimicrobial resistance and conjugative plasmid replicons, respectively. SCC*mec*Finder 1.2 was used for Staphylococcal Cassette Chromosome *mec* (SCC*mec*) typing [40, 45]. MLST 2.0 was used to determine sequencing types [40, 41, 46].

The contigs were subjected to single nucleotide polymorphism (SNP) analysis using CSIPhylogeny [47] with a minimal Z-score of 1.96 and a minimal depth at SNP position of 10x. *S*. *pseudintermedius* DG072 (accession number SAMN17102122) and *S*. *felis* FDAARGOS_1014 (accession number SAMN16357183) were used as references for SNP analysis. Sequenced genomes were subjected to whole-genome MLST (wgMLST) analysis using Cano-wgMLST _BacCompare [48, 49]. MRSP strains from recent studies conducted in the same city were included for genetic comparison purpose [12, 13]. Isolates of *S*. *felis* included in the present study were downloaded from the Bacterial and Viral Bioinformatics Resource Center (https://www.bv-brc.org/) and from previous studies [37, 50, 51] (S2 Table in S1 File). All trees were generated using the iTOL online software and midpoint rooting [36].

### Statistical analyses

Associations between the isolation of specific *Staphylococcus* spp, including MRS, MDR, and MRSP, and various factors was evaluated via univariate analysis using Fisher’s exact test. These factors included the origin of the animals (hospitalized vs. non-hospitalized), use of antimicrobials, sex, contact with other animals, outdoor access, and presence of comorbidities. Associations were expressed as odds ratios (ORs) and 95% confidence intervals (CIs), and statistical significance was set at *p* < 0.05. To conduct statistical analyses for age, the animals were categorized into kittens, adults, and elderly population as described by Santana et al. [13]. All analyses were performed using Stata version 14 (Stata Corp. LLC, USA).

## Results

### Samples and epidemiological data

A total of 109 cats were sampled, including 51.4% (56/109) females who were mostly adults (average age of 58 months ± 47 months) with outdoor access (34.9%) and interacted with other animals (82.6%; S1 Table in S1 File). Approximately 17.4% exhibited a history of antimicrobial use, primarily of cephalothin (36.8%), ceftriaxone (31.6%), metronidazole (21.1%), and clindamycin (21.1%), in the last six months. Approximately 36.7% of the cats exhibited at least one comorbidity.

### Frequency of *Staphylococcus* spp.

**Fig 1:**
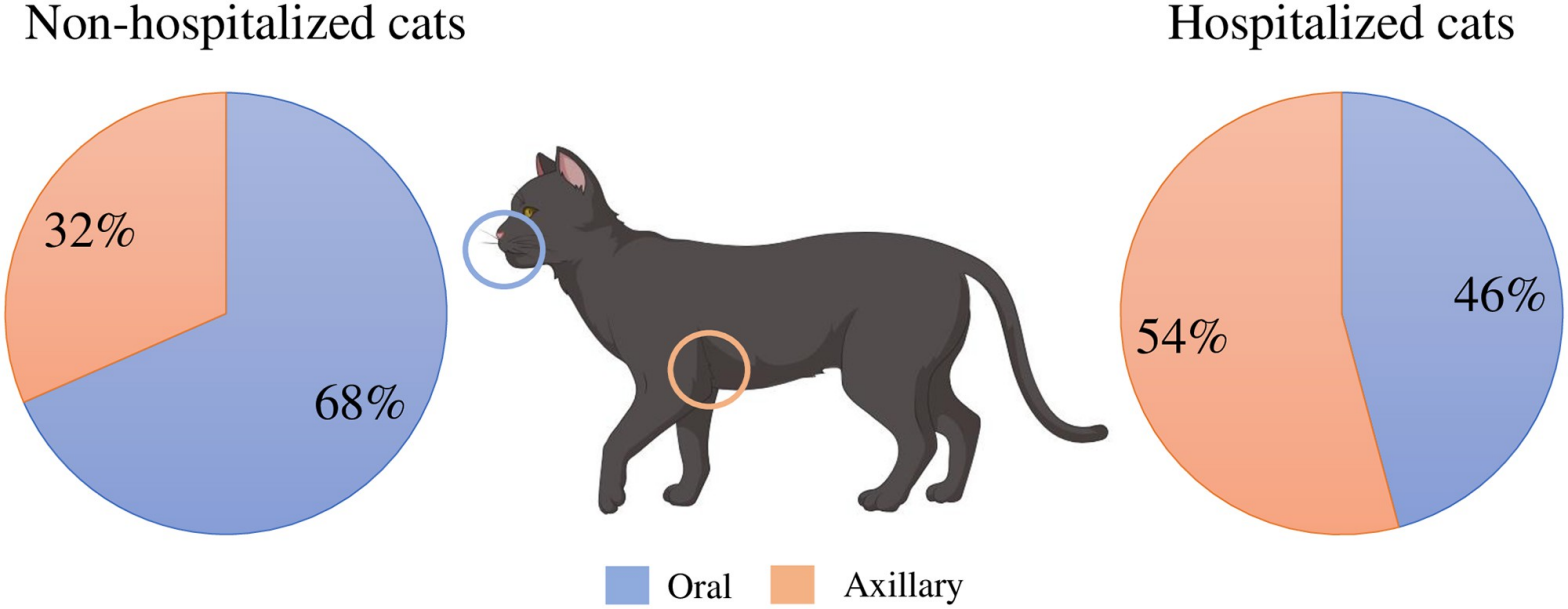
Distribution of *Staphylococcus* spp. in cats based on the isolation site and category.

A total of 81 *Staphylococcus* spp. isolates, including 70.4% (57/81) from non-hospitalized cats and 29.6% (24/81) from hospitalized cats (Table 1), were analyzed in this study. Thirty-one isolates (38.3%) were obtained from the axillary site and 50 (61.7%) from the oral site (Fig 1).

**Table 1:** Distribution of *Staphylococcus* spp. based on the type of cats sampled and isolation site.

Isolated species	Non-hospitalized cats			Hospitalized cats			Total (%)
	Oral (%)	Axillary (%)	Total (%)	Oral (%)	Axillary (%)	Total (%)	
*S*. *felis*	15 (38.5)	1 (5.5)	16 (28.1)	2 (18.2)	2 (15.4)	4 (16,7)	20 (24.7)
*S*. *epidermidis*	5 (12.8)	5 (28.1)	10 (17.5)	0 (0.0)	1 (7.7)	1 (4.2)	11 (13.6)
*S*. pseudintermedius*	0 (0.0)	2 (11.1)	2 (3.5)	3 (27.2)	3 (23)	**6 (25)** ^1^	8 (10)
*S*. *haemolyticus*	1 (2.6)	2 (11.1)	3 (5.4)	2 (18.2)	2 (15.4)	4 (16,7)	7 (8.6)
*S*. *aureus*	6 (15.3)	0 (0.0)	6 (10.5)	1 (9.1)	0 (0.0)	1 (4.2)	7 (8.6)
*S*. *capitis*	3 (7.7)	2 (11.1)	5 (8.8)	0 (0.0)	0 (0.0)	0 (0.0)	5 (6.2)
*S*. *xylosus*	2 (5.1)	2 (11.1)	4 (7.0)	1 (9.1)	0 (0.0)	1 (4.2)	5 (6.2)
*S*. *warneri*	2 (5.1)	1 (5.5)	3 (5.4)	0 (0.0)	1 (7.7)	1 (4.2)	4 (4.9)
*S*. *pettenkoferi*	1 (2.6)	0 (0.0)	1 (1.7)	1 (9.1)	1 (7.7)	2 (8)	3 (3.7)
*S*. *pseudoxylosus*	0 (0.0)	1 (5.5)	1 (1.7)	0 (0.0)	1 (7.7)	1 (4.2)	2 (2.5)
*S*. *simulans*	2 (5.1)	0 (0.0)	2 (3.5)	0 (0.0)	0 (0.0)	0 (0.0)	2 (2.5)
*S*. *lentus*	1 (2.6)	1 (5.5)	2 (3.5)	0 (0.0)	0 (0.0)	0 (0.0)	2 (2.5)
*S*. *saprophyticus*	0 (0.0)	0 (0.0)	0 (0.0)	0 (0.0)	1 (7.7)	1 (4.2)	1 (1.2)
*S*. *nepalensis*	0 (0.0)	0 (0.0)	0 (0.0)	1 (9.1)	0 (0.0)	1 (4.2)	1 (1.2)
*S*. *hominis*	0 (0.0)	0 (0.0)	0 (0.0)	0 (0.0)	1 (7.7)	1 (4.2)	1 (1.2)
*S*. *lugnudensis*	0 (0.0)	1 (5.5)	1 (1.7)	0 (0.0)	0 (0.0)	0 (0.0)	1 (1.2)
*S*. *sciuri*	1 (2.6)	0 (0.0)	1 (1.7)	0 (0.0)	0 (0.0)	0 (0.0)	1 (1.2)
**Total (%)**	39 (100)	18 (100)	57 (100)	11 (100)	13 (100)	24 (100)	81 (100)

Statistical significance was set at 5% level.

*S. *pseudintermedius* was the only representative of the *S*. *intermedius* group (SIG) group identified in this study.

^**1**^*S*. *pseudintermedius* frequency was higher in the hospitalized cats than in the non-hospitalized cats (*p* = 0.007).

Of the 81 isolates obtained, 65 (80.3%) were identified via MALDI-TOF or PCR for SIG group differentiation. Sixteen isolates (19.7%) were subjected to sequencing of *rpo*B and *16S rRNA* due to their low identity scores in MALDI-TOF MS. *S*. *felis* was the most commonly detected species in this study (24.7%; Table 2), followed by *S*. *epidermidis* (13.6%), *S*. *pseudintermedius* (10%), and *S*. *haemolyticus* (8.6%). *S*. *pseudintermedius* frequency was higher in hospitalized cats than in the non-hospitalized cats (*p* = 0.007). *S*. *pseudintermedius* was the only representative species of SIG group identified in this study.

**Table 2:** Distribution of methicillin-resistant *Staphylococcus* (MRS, *mec*A-positive) strains in non-hospitalized and hospitalized cats of Belo Horizonte, Minas Gerais.

Species	Non-hospitalized cats (%)	Hospitalized cats (%)	Total (%)
*S*. *pseudintermedius*	0 (0.0)	5 (55.5)	5 (33.3)
*S*. *haemolyticus*	1 (16.6)	3 (33.4)	4 (26.7)
*S*. *epidermidis*	1 (16.6)	1 (11.1)	2 (13.3)
*S*. *lentus*	2 (33.6)	0 (0.0)	2 (13.3)
*S*. *pettenkoferi*	1 (16.6)	0 (0.0)	1 (6.7)
*S*. *capitis*	1 (16.6)	0 (0.0)	1 (6.7)
**Total (%)**	6 (100)	9 (100)	15 (100)

### Antimicrobial sensitivity

**Fig 2:**
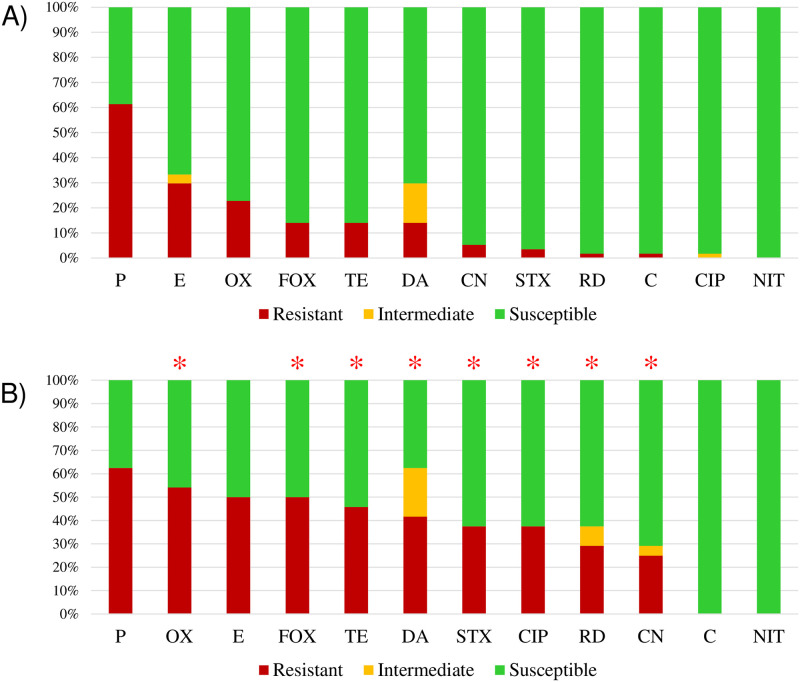
Antimicrobial resistance profiles of isolates from non-hospitalized (A) and hospitalized (B) cats. *Statistical significance at 5% level. Resistant isolates from hospitalized and non-hospitalized cats were compared using Fisher’s exact test. OX, oxacillin; FOX, cefoxitin; P, penicillin; CIP, ciprofloxacin; C, chloramphenicol; DA, clindamycin; E, erythromycin; CN, gentamicin; NIT, nitrofurantoin; RD, rifampicin; STX, sulfamethoxazole-trimethoprim; TE, tetracycline.

**Fig 3:**
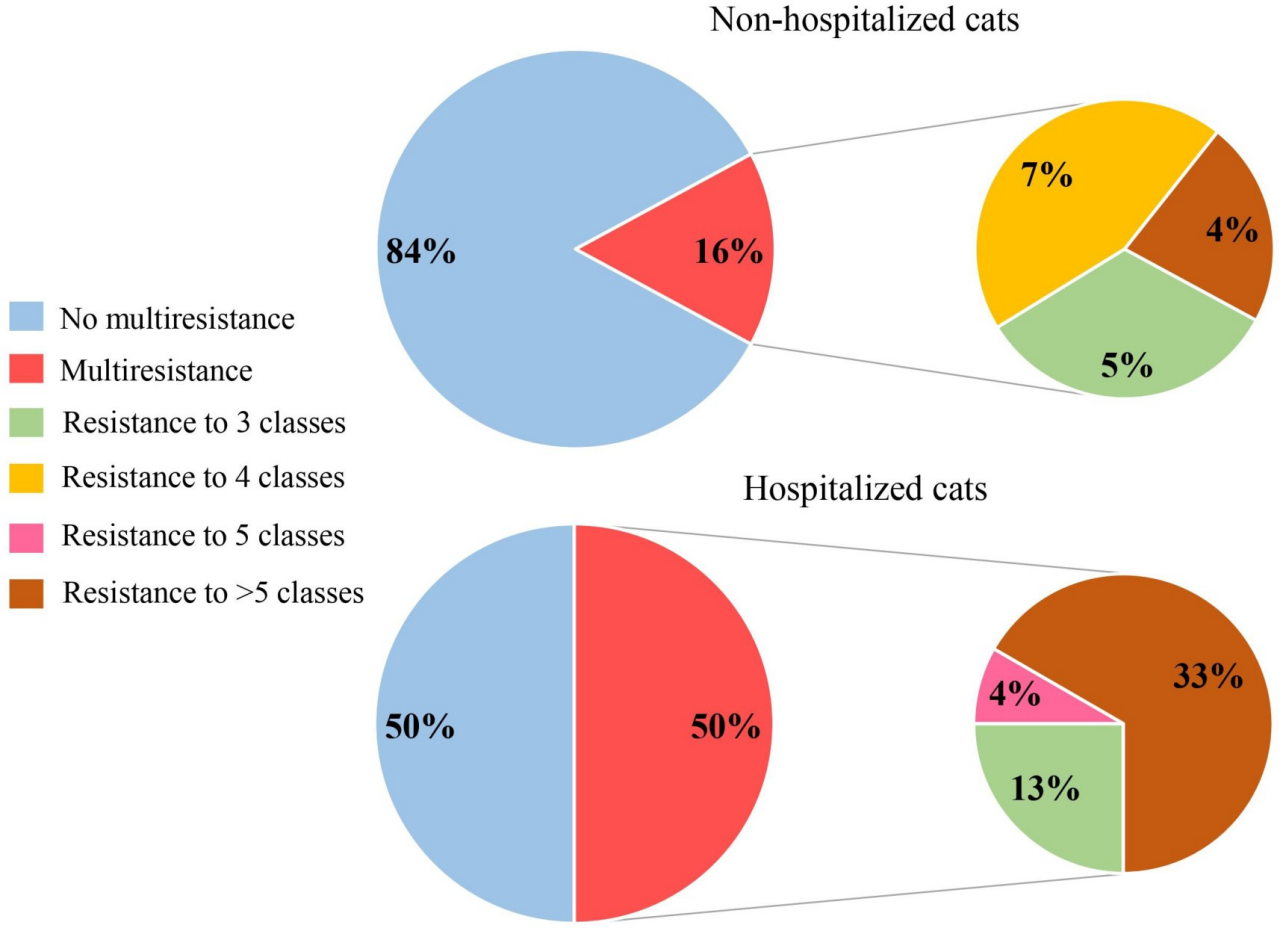
Percentages of multidrug-resistant staphylococcal isolates obtained from the non-hospitalized and hospitalized cats in Belo Horizonte, Brazil between June and October, 2022.

Most *Staphylococcus* spp. isolates were resistant to penicillin (61.7%; Fig 2) at a frequency higher than that observed for other antimicrobials (*p* = 0.0001), followed by oxacillin (35.8%) and erythromycin (32.1%; Table 3). Resistance to sulfamethoxazole-trimethoprim, ciprofloxacin, and rifampicin was higher in the hospitalized cats (Fig 2). Twenty-one isolates (26%) were classified as MDR, and hospitalized animals were almost 4-times more likely to be carriers of MDR *Staphylococcus* than the non-hospitalized animals (*p* = 0.039; OR = 3.37; 95% CI: 1.01–11.24; Fig 3). The incidence of MDR was high in *S*. *pseudintermedius* (28.6%; *p* = 0.0032; OR = 11.6; 95% CI: 1.77–123.62), whereas most *S*. *felis* strains (65%) were sensitive to all tested antimicrobials.

**Table 3:** Variables associated with methicillin-resistant *Staphylococci* (MRS) infection in domestic cats.

Variable	MRS*	*p-value*	*Odds ratio*
Sex			
Females	4/52	*p = 0*.*293*	-
Males	7/46		
Agre group **			
<12 months (A)	1/22	*p = 0*.*684*	-
≥12 and <84 months (B)	5/56	*p = 0*.*877*	
≥84 months (C)	4/29	*p = 0*.*335*	
Contact with other animals**			
Yes	8/82	*p = 0*.*949*	-
No	1/11		
Outdoors access**			
Yes	6/32	*p = 0*.*094*	-
No	4/64		
**Comorbidities**			
Yes	9/31	***p = 0*.*002***	**9.73**
No	2/67		(CI: 1.81–95.61)
**Hospitalization**			
Yes	7/25	***p = 0*.*0141***	**5.11**
No	4/73		(CI: 1.16–25.42)
**Use of antimicrobials** **			
Yes	8/11	***p = 0*.*0001***	**20.85**
No	3/86		(CI: 4.3–133.29)

*Per animal;

**Excluding not informed (NI) data;

CI = confidence interval.

**Fig 4:**
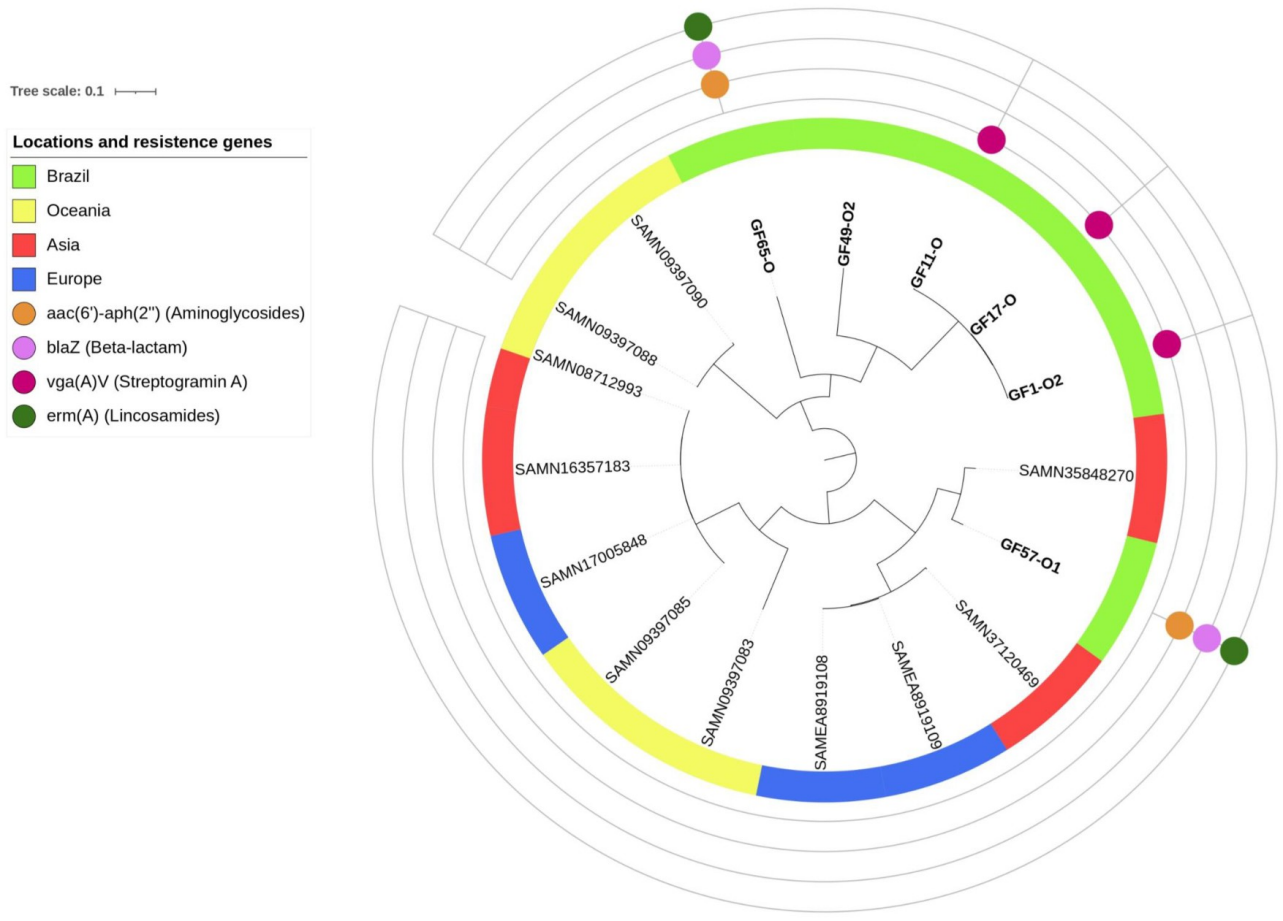
Phylogenetic tree constructed based on single nucleotide polymorphism (SNP) analysis of *S*. *felis* isolates in this study (GF) using CSIPhylogeny. Tree image was generated using iTOL online, with midpoint rooting.

Six *S*. *felis* isolates were subjected to genomic sequencing. The analysis confirmed the presence of genes resistant to lincosamides (*ermA*), penicillins (*blaZ*), streptogramin A (*vga(A)*), and aminoglycosides (*aac(6’)-aph(2”)*). However, these genes have not been detected in *S*. *felis* genomes analyzed in previous studies (Fig 4) [37, 50–52].

Three isolates detected in this study (GF1-O2, GF17-O, and GF11-O) showed high similarities, with 11–80 SNPs. In SNP analysis, these three isolates, along with two other isolates from this study, were clustered in the same clade (Fig 4). The last isolate from this study was clustered in another clade and grouped with *S*. *felis* strains previously isolated from Europe and Asia (Fig 4) [50, 51].

**Fig 5:**
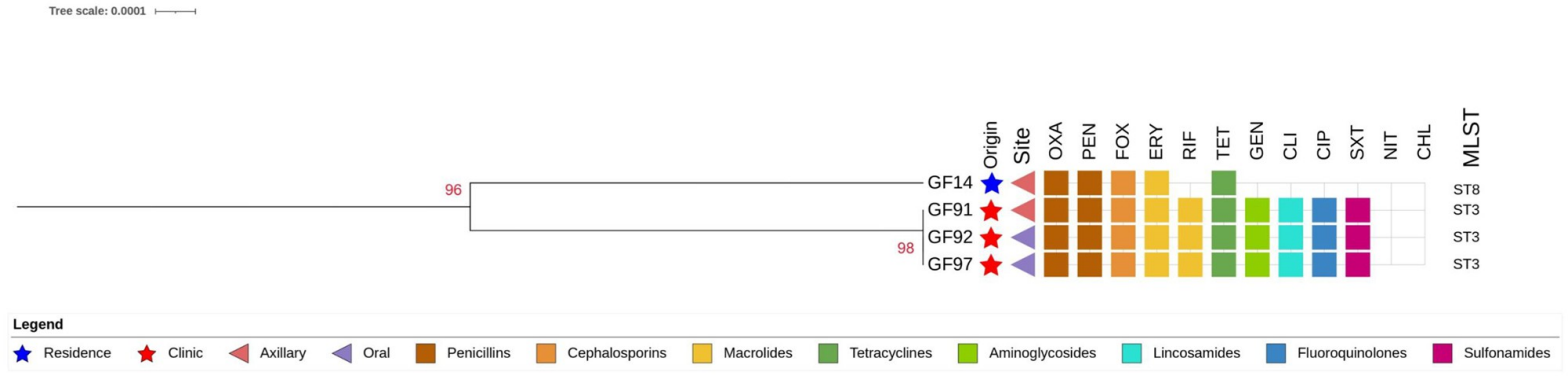
Phylogenetic tree constructed based on the analysis of the seven concatenated genes identified via multilocus sequence typing (MLST) of *Staphylococcus haemolyticus*. Red stars indicate the isolates from hospitalized cats (clinical). Blue stars indicate the isolates from non-hospitalized cats (domestic). Triangles indicate the sites at which the isolates were detected, and colored squares indicate the antimicrobial resistance profiles. Tree image was generated using iTOL online, with midpoint rooting.

**Fig 6:**
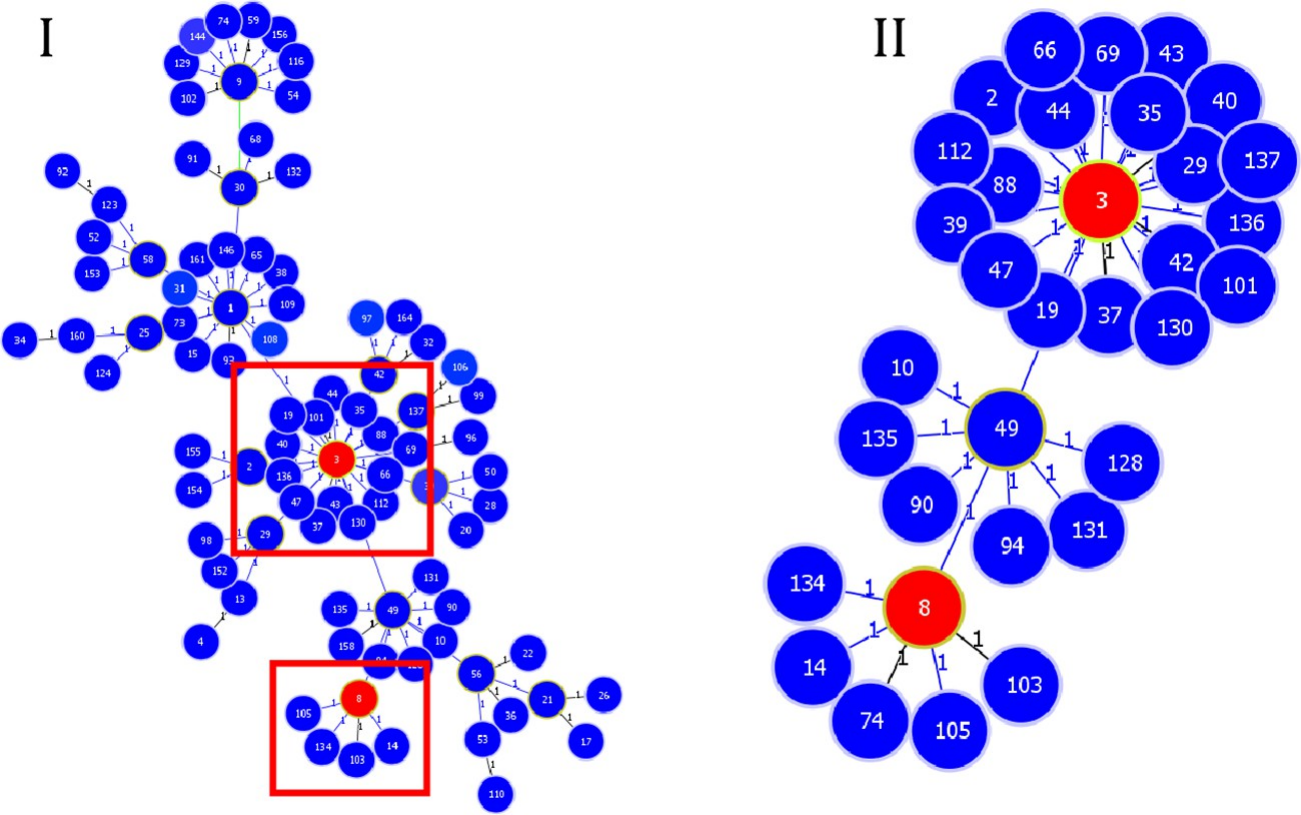
Population structure of *S*. *haemolyticus* based on a single locus variant. In section I, sequence types (STs) deposited into the PubMLST database are shown in blue, whereas those detected in this study are shown in red. In section II, clonal complex (CC)-3 was observed, with ST3 being the founder, and ST8 detected after two alleles. Tree image was generated using the PHYLOViZ software 2.0.

A total of 10.4 and 25% of the non-hospitalized and hospitalized cats, respectively, tested positive on MRS (*p* = 0.0141). Fifteen isolates (18.5%) were positive for *mec*A (Table 2). Methicillin resistance was associated with *S*. *pseudintermedius* (*p* = 0.0046; OR = 10.5; 95% CI: 1.66–74.95). Among these, four strains of MRSH (26.7%) were isolated from three hospitalized cats and one domestic cat. In MLST analysis, the MRSH isolates were classified into two STs: ST3 (all isolates from hospitalized cats) and ST8 (all isolates from non-hospitalized cats; Figs 5 and 6).

**Fig 7:**
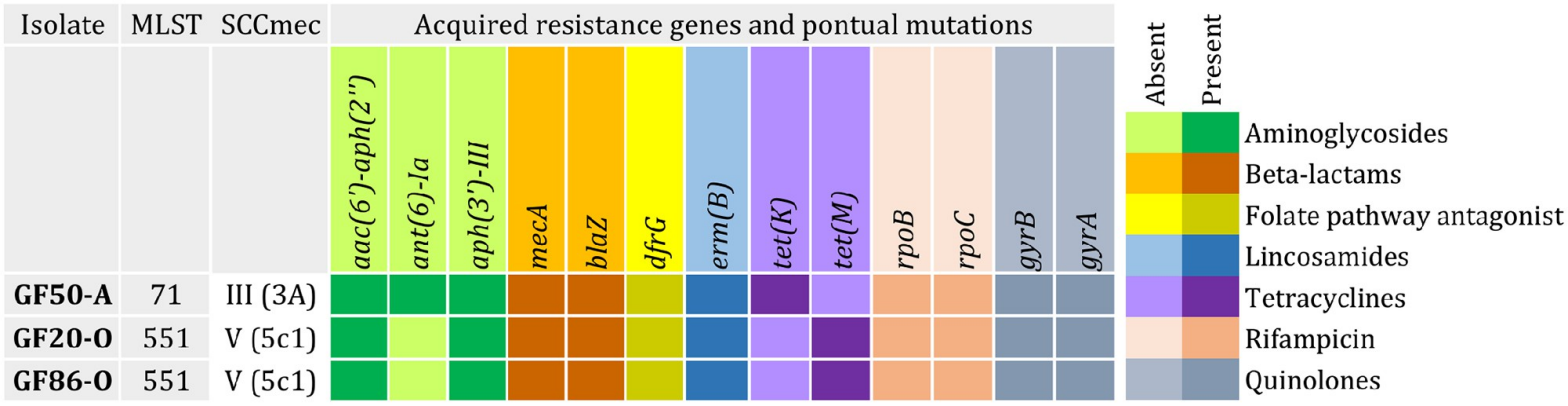
MLST and staphylococcal cassette chromosome *mec* (SCC*mec*) typing results and resistance genes and point mutations identified in three MRSP (GF) isolates from hospitalized cats in Belo Horizonte, Minas Gerais.

**Fig 8:**
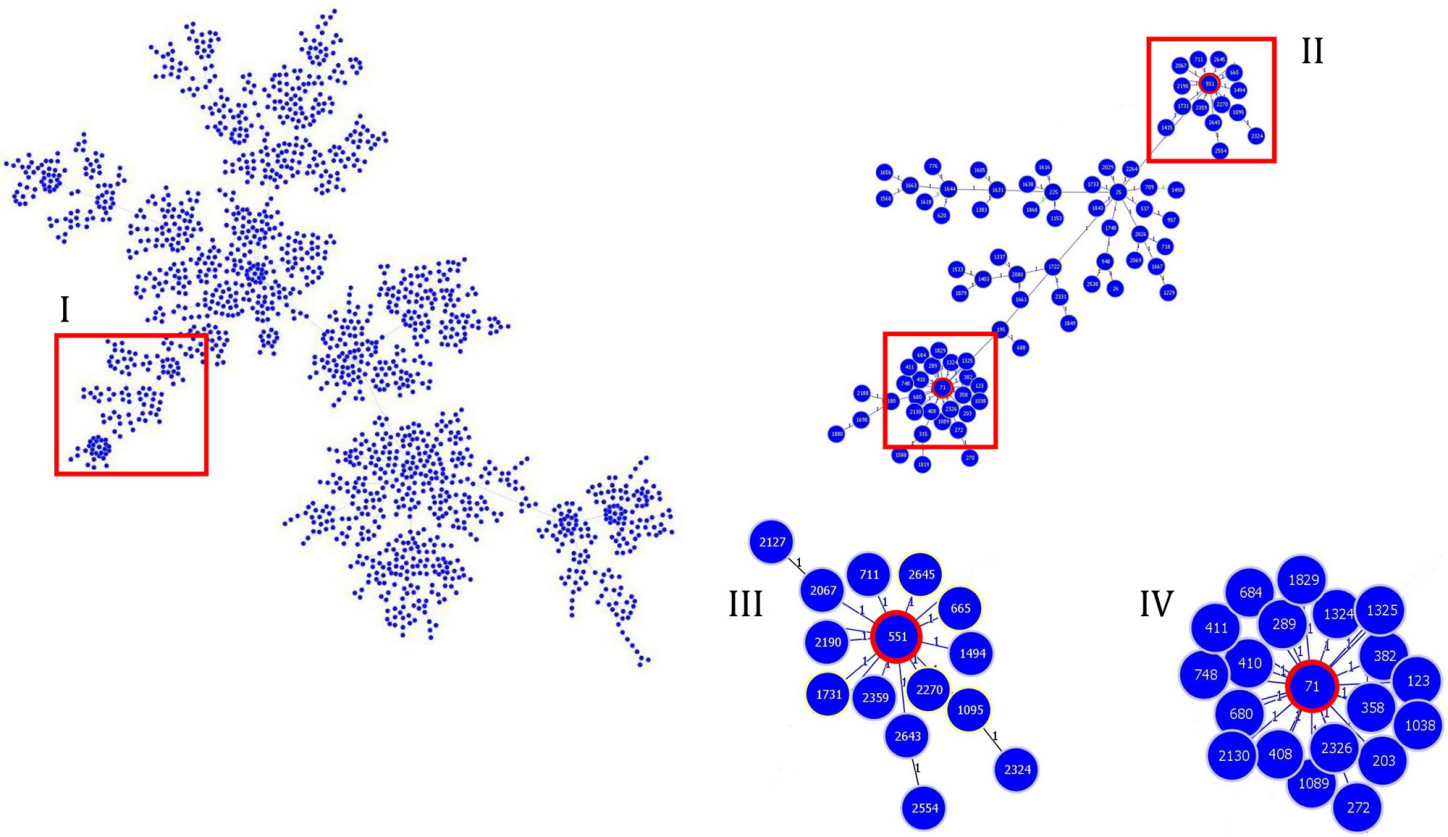
Population structure of *S*. *pseudintermedius* based on a single locus variant. Sections I and II show the clusters of STs where ST551 and ST71 were detected. Sections III and IV correspond to CC551 and CC71, respectively. Tree image was generated using the PHYLOViZ software.

**Fig 9:**
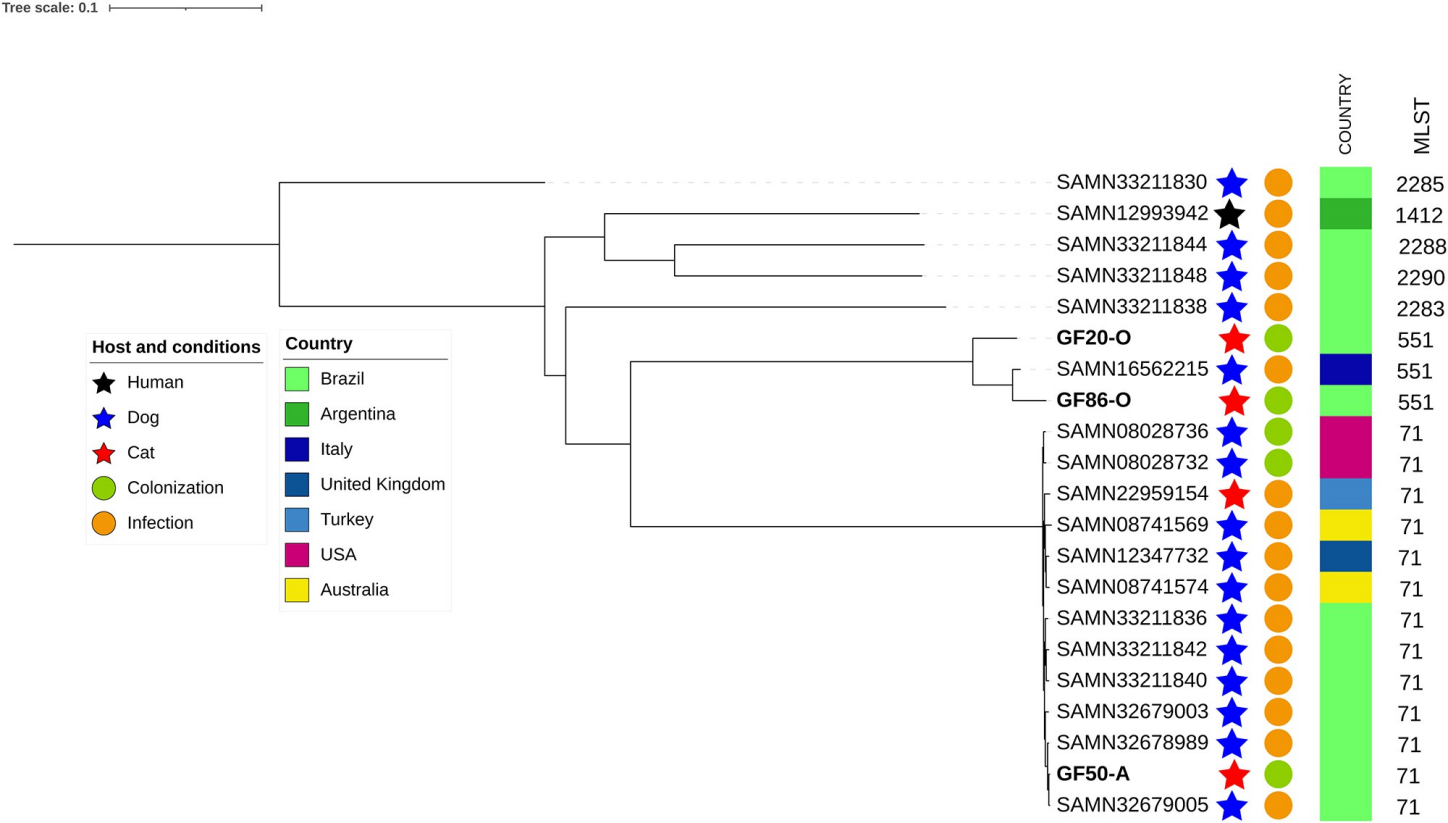
Phylogenetic tree constructed based on SNP analysis of the MRSP (GF) isolates detected in this study. MRSP isolates from previous studies in Brazil [12, 13] and other countries were used for similarity assessment. Tree image was generated using iTOL online, with midpoint rooting.

Five MRSP isolates were identified in this study from three hospitalized cats. Three isolates (one from each animal) were selected and subjected to WGS analysis. In MLST analysis, two well-known STs were detected: ST551, the founder of CC551, and ST71, the founder of CC71, in one isolate (Figs 7 and 8). MRSP isolates were compared using wgMLST and SNP analyses. Both analyses dismissed the hypothesis of clonality between the two isolates classified as ST551 (1354 SNPs and 281 alleles in wgMLST). However, MRSP ST71 showed similarity with isolates previously detected in Brazil [12, 13] from dogs with MRSP infections, especially with an isolate from a surgical site infection, BR19 (accession number SAMN32679005), which differed by only eight SNPs (Fig 9). Interestingly, the two STs detected in this study (ST551 and ST71) exhibited differences in their resistance genes. Specifically, ST551 harbored *tet(M)* that confers tetracycline resistance, whereas ST71 harbored *tet(K)*. Additionally, ST71 alone harbored the *ant(6)-Ia* gene that confers resistance to aminoglycosides (Fig 7). However, both STs shared several resistance genes and point mutations associated with quinolones, rifampicin, lincosamides, and folate pathway antagonists.

### Factors associated with MRS colonization

Cats with a history of antimicrobial treatment were almost 21-times more likely to harbor MRS. Hospitalized animals and those with comorbidities, regardless of hospitalization, were almost six- and ten-fold more likely to acquire MRS, respectively (Table 3).

## Discussion

### Antimicrobial resistance of *Staphylococcus* spp.

*S*. *felis*, the representative *Staphylococcus* species infecting domestic cats [17, 53], was the most commonly isolated species in this study. Other species found in non-hospitalized cats, such as *S*. *epidermidis*, *S*. *aureus*, and *S*. *capitis*, also colonized the healthy cats, suggesting that these microbes are part of the natural microbiota of cats [17, 54, 55].

To date, only a few studies have genetically evaluated S. *felis* isolates, with none focused in Brazil. In this study, seven isolates of S. *felis* from different individuals were subjected to genomic sequencing. Similarity analysis revealed that most isolates were grouped together, indicating a higher degree of similarity among them compared to that among the isolates from other countries. This resemblance also indicates evolution from a common ancestor, accounting for the detection of resistance genes in many Brazilian isolates. These findings are in contrast to previous reports on the absence of these determinants [37, 50, 51]. Therefore, *S*. *felis* strains exhibit a distinct epidemiology in Brazil, easily acquiring multidrug resistance genes. However, further studies are necessary to understand the epidemiological characteristics and extent of evolution of such multidrug resistance in Brazilian *S*. *felis* strains.

Here, we observed the association between hospitalized cats and isolation of *S*. *pseudintermedius*. This finding is important as *S*. *pseudintermedius* is a common colonizer of both dogs and cats associated with skin diseases and surgical wounds [12, 13, 56]. Interestingly, *S*. *pseudintermedius* isolates were approximately 11-times more likely to be MRS compared to the other *Staphylococcus* spp., consistent with previous reports [12, 13, 17, 22, 57].

In contrast to the isolates of *S*. *pseudintermedius*, *S*. *felis* isolates exhibited high sensitivity to most of the tested antimicrobials, with nearly two-thirds of the *S*. *felis* isolates being sensitive to all tested compounds. These findings suggest that the use of antimicrobials and hospital environment cause a possible shift in microbiota, decreasing the prevalence of *S*. *felis*, which is sensitive to most antibiotics, but increasing that of MRS, especially *S*. *pseudintermedius*, consistent with previous reports [17, 22, 58]. This finding was further supported by MRS frequency in hospitalized vs. non-hospitalized cats (25 vs. 10.4%; *p* = 0.0141).

### Molecular characterization of MRSP isolates

Studies evaluating *Staphylococcus* isolates in cats are scarce, with no study characterizing MRSP isolates in Brazil to date. In this study, one MRSP per animal (n = 3) was selected and subjected to genomic sequencing. Two STs were identified, ST551 (two isolates) and ST71. ST71 is distributed worldwide, shows significant antimicrobial resistance, and mainly infects dogs, occasionally infecting other species, such as cats and humans [12, 22, 59–61]. Similar to previous studies in Brazil, ST71 isolate harbored SCC*mec* IIIa, which is common among CC71 isolates [13, 62].

The other two isolates were classified as ST551, the founder of CC551. Although less common than the isolates from CC71, CC551 isolates also exhibit resistance to antimicrobials and infection ability [22, 63]. In contrast to the CC71 isolates, CC551 isolates commonly harbor SCC*mec* Vc (5c1) [11, 64, 65], which is consistent with our findings. Both CC71 and CC551 are distinguished by their high antimicrobial resistance and association with animals in veterinary clinics [11, 13, 22, 62]. Here, both STs harbored genes and specific point mutations conferring resistance to various non-beta-lactam antibiotics, albeit with notable differences, particularly for tetracycline and aminoglycosides. This observation is expected considering the distinct genetic backgrounds of strains from different CCs, which can lead to varying antibiotic resistance profiles [8, 9, 66], including the SCC*mec* types observed in this study.

As the three MRSP isolates showed the same phenotypic resistance patterns, we hypothesized that they represented the same clone transmitted within the hospital environment, as indicated in previous studies on dogs [10, 13, 67]. Our hypothesis was supported by the fact that the two strains had the same STs and resistance gene patterns. However, our SNPs and wgMLST results rejected this hypothesis, suggesting that the MRSP strains originated from different sources. These findings further confirm the potential of MRSP transmission in clinics, as reported in previous studies [11, 56].

ST71 isolate is similar to other reported MRSP strains in Brazil colonizing and infecting dogs in the same clinical environment (Fig 9) [12, 13, 62]. This indicates the possible transmission of MRSP from dogs to hospitalized cats [10, 13, 68], which is insignificant to the context of this study in cats. Transmission of isolates in veterinary environments primarily occurs during animal handling [12, 13, 22, 69], underscoring the need to re-evaluate the protocols used in veterinary clinics.

### Molecular characterization MRSH isolates

In addition to the MRS isolates, other methicillin-resistant species of clinical importance, such as *S*. *haemolyticus*, were detected in this study. Isolation of this species from hospitalized cats is associated with transmission during hospital stay. Notably, MRSH is rarely observed in non-hospitalized cats [60, 62] but commonly causes hospital-acquired infections in humans [14, 15, 18, 70–72] and occasionally in hospitalized cats and dogs [16, 17, 19, 73, 74].

The three MRSH isolates identified in this study were subjected to MLST. The isolate from the only non-hospitalized cat that tested positive for MRSH was classified as ST8, an ST previously reported in humans, animals, and hospitals [20, 33, 75, 76]. This cat appeared healthy with no history of antimicrobial use but had outdoor access. In contrast, the isolates from hospitalized cats were classified as ST3, the founder of CC3, which is the main and most widespread CC of MRSH worldwide that commonly infects humans and domestic animals [14, 74, 77]. The three isolates from hospitalized animals receiving antibiotic therapy showed the same phenotypic resistance profiles and were isolated within a short period (within nine days). This highlights the possible spread of MRSH strains among cats in hospitals [74, 78, 79]; further genomic sequencing of the detected isolates is necessary for validation.

### Clinical and epidemiological factors associated with MRS and MRSP colonization

In this study, antimicrobial use, comorbidities, and hospitalization were associated with MRS isolation. Several studies have highlighted veterinary hospitalization as a determining factor for MRS infections due to the intensive use of antimicrobials to treat hospitalized animals [2, 12, 13]. This study focused solely on the MRS colonization of animals and did not assess MRSP infection. However, MRSP isolates also exhibit great potential for dissemination and infection, possibly causing hospital outbreaks [10, 13]. Overall, our findings suggest that cats play crucial roles in the dissemination of MRS, including MRSP, and associated infections in clinical settings, highlighting the need for effective surveillance protocols and rational use of antimicrobials in veterinary hospitals and clinics.

## Supporting information

S1 File (DOCX)
