# Supplementary material for: Occurrence, genetic diversity, and antimicrobial resistance of methicillin-resistant Staphylococcus spp. in hospitalized and non-hospitalized cats in Brazil
Source: PLoS One. 2024 Oct 3;19(10):e0309711. doi: 10.1371/journal.pone.0309711 (PMC11449366; doi:10.1371/journal.pone.0309711)
Supplement: S1 File — (DOCX) [file pone.0309711.s001.docx]

**Supplementary Material**

**Supplementary Table 1.** Epidemiological Data of Sampled Cats by Group

| **General Data** | | Non-Hospitalized Cats (%) | Hospitalized Cats (%) | Total (%) |
| --- | --- | --- | --- | --- |
| Females | | 44 (57.1%) | 12 (37.5%) | 56 (51.3%) |
| Males | | 33 (42.8%) | 20 (62.5%) | 53 (48.6%) |
| Age group | <12 months | 16 (20.8) | 6 (18.7) | 22 (20.2) |
|  | ≥12 <84 months | 40 (51.9) | 16 (50) | 56 (51.3) |
|  | ≥84 months | 21 (27.2) | 8 (25) | 29 (26.6) |
| Outdoor access | | 23 (21.1%) | 15 (46.8%) | 38 (34.9%) |
| Interaction with other animals | | 73 (94.8%) | 17 (53.1%) | 90 (82.5%) |
| Comorbidities | | 11 (14.2%) | 29 (90.6%) | 40 (36.6%) |
| History of antimicrobial therapy | | 4 (5.1%) | 15 (46.8%) | 19 (17.4%) |

*Excluding 'Not Informed data. Animals were categorized into kittens, adults and elderly according to [41].

**Supplementary Table 2.** MRSP isolates from the present study and from previous brazilian studies used for similarity assessment, with their respective accession numbers. In addition, *S. felis* isolates from the present study and their respective accession numbers.

| **Isolate** | **Organism** | **Source (Body site)** | **Genome Accession** | **Reference** |
| --- | --- | --- | --- | --- |
| GF50-A | MRSP | Colonization (axilla) | SAMN40436489 | This study |
| GF20-O | MRSP | Colonization (oral) | SAMN40436490 |  |
| GF86-O | MRSP |  | SAMN40436491 |  |
| BR19 | MRSP | Infection (SSI) | SAMN32679005 | Viegas et al., 2022 |
| LB1610 | MRSP | Infection (pyoderma) | SAMN33211848 | Teixeira et al., 2023 |
| LB1611 | MRSP |  | SAMN33211830 |  |
| LB1625 | MRSP |  | SAMN33211836 |  |
| LB1630 | MRSP |  | SAMN33211838 |  |
| LB1635 | MRSP |  | SAMN33211840 |  |
| LB1674 | MRSP |  | SAMN33211842 |  |
| LB1683 | MRSP |  | SAMN33211844 |  |
| GF1-O2 | *S. felis* | Colonization (oral) | SAMN40436492 | This study |
| GF11-O | *S. felis* |  | SAMN40436493 |  |
| GF17-O | *S. felis* |  | SAMN40436494 |  |
| GF49-O2 | *S. felis* |  | SAMN40436495 |  |
| GF57-O1 | *S. felis* |  | SAMN40436496 |  |
| GF65-O | *S. felis* |  | SAMN40436497 |  |
